# Nature of the Heart’s Contraction

**Published:** 1866-10

**Authors:** 


NATURE OF THE HEART'S CONTRACTION.—M. Marey has been studying the action of the muscles of the heart in comparison with that of the ordinary muscles, and has communicated the results of his researches to the French Academy. The contraction of the muscular fibres of the heart, like those of the bands of non-striated muscle, are continuous, and not vibratory like those of the ordinary muscles. In order to establish a relation between the phenomena of contraction in the two sorts of muscular tissue, M. Marey says that a ventricular systole, however slow, corresponds to a single muscular contraction, and that thus a systole holds the same relation to a contraction that a muscular vibration (with ordinary muscles) does to the peculiar sound or musical note it produces.—*Lancet.*

